# Modest sexual size dimorphism and allometric growth: a study based on growth and gonad development in the wolf spider *Pardosa pseudoannulata* (Araneae: Lycosidae)

**DOI:** 10.1242/bio.058771

**Published:** 2021-12-10

**Authors:** Fan Zhang, Xiaoqiong Chen, Chi Zeng, Lelei Wen, Yao Zhao, Yu Peng

**Affiliations:** 1 Hubei Key Laboratory of Regional Development and Environmental Response, Faculty of Resources and Environmental Science, Hubei University, Wuhan 430062, China; 2 State Key Laboratory of Biocatalysis and Enzyme Engineering, School of Life Sciences, Hubei University, Wuhan 430062, China; 3 Hubei Key Laboratory of Quality Control of Characteristic Fruits and Vegetables, College of Life Science and Technology, Hubei Engineering University, Xiaogan 432000, China

**Keywords:** Sexual size dimorphism, Wolf spider, Growth plasticity, Allometric growth

## Abstract

Sexual size dimorphism (SSD) is a notable phenomenon in terrestrial animals, and it is correlated with unusual morphological traits. To date, the underlying sex-specific growth strategies throughout the ontogenetic stage of spiders are poorly understood. Here, we comprehensively investigated how the growth trajectories and gonad development shaped SSD in the wolf spider *Pardosa pseudoannulata* (Araneae: Lycosidae)*.* We also hypothesized the potential growth allometry among the carapace, abdomen, and gonads of spiders in both sexes. By measuring the size of the carapace and abdomen, investigating developmental duration and growth rate, describing the gonadal sections, and calculating the area of gonads at all instars from hatching to maturity, we demonstrated that SSD results from sex-specific growth strategies. Our results indicated that the growth and developmental differences between both sexes appeared at early life stages, and there was allometric growth in the carapace, abdomen, and gonads between males and females.

## Abstract (precis)

**Summary:** Our findings indicated that growth and developmental differences between sexes appeared at early life stages, and there was allometric growth in the carapace, abdomen, and gonads between males and females.

## INTRODUCTION

Females and males of most animals differ dramatically in their body size, a morphological syndrome called sexual size dimorphism (SSD) (Fairbairn, 1997). Sexual size dimorphism is ubiquitous in the animal kingdom (Hedrick and Temeles, 1989). In many birds and mammals, males are usually larger than females (Cabana et al., 1982; Ralls, 1977; Szekely et al., 2004), whereas females are the larger sex in most ectotherms (Arak, 1988; Shine, 1989). Compared with some male-biased SSD that tend to be extreme (Norman et al., 2010), female-biased SSD is far more common. For instance, the most extreme female gigantism is found in orb-weaving spiders from the clade Araneoidea (Foellmer and Moya-Laraño, 2007). Spiders comprise the most remarkable SSD among terrestrial animals (Kuntner and Coddington, 2020) and are an excellent group in which to study various aspects of SSD.

SSD in animals is generally attributed to differential pressures of natural selection and sexual selection on females and males (Darwin, 1871). These sex-differential selection pressures are related to reproductive roles, life styles, and mating behaviour (Fairbairn, 2007). In many arthropods, females grow larger than males, which are often explained by selection pressures (Blanckenhorn, 2005). Males typically devote their resources to sperm production, searching for mates, male–male competition, and escape of cannibalism (Kralj-Fišer et al., 2013). Females, on the other hand, invest large amounts of resources in their offspring, including egg-laying and hatching. To gain advantages in these selections, females and males must have evolved sex-specific growth strategies (Andersson, 1994). However, to truly understand the sex-specific growth strategies requires a complete analysis of the entire development of both sexes. Quantifying the relationship between adult size and developmental duration does not reveal the period of sex-specific growth. To date, a lifelong analysis across growth patterns in spiders is rare (Rohner et al., 2017), presumably due to lack of diagnostic genetic or morphological markers about the sex of juvenile spiders.

SSD can easily be certified in adult spiders by morphological features. Morphological differences between sexes first appear in subadult spiders (i.e. one moult to mature). The end of the subadult male pedipalps are swollen and specialized into the palp organs before maturation. Through observation of bulb development inside the tip of pedipalp in *Parasteatoda tepidariorum*, previous studies have found that the anlagen of the bulb already exists in the distal tip of the male pedipalps in the pre-subadult stage (Quade et al., 2019). Similarly, the epigyne of subadult females is already visible below the cuticle in some spiders (Biaggio et al., 2016). Other studies on *Pholcus phalangioides* (Michalik and Uhl, 2005) spermatogenesis and the *Loxosceles intermedia* (Margraf et al., 2011) genetic system have suggested that the male reproductive system must have developed before the subadult stage. These morphological and physiology analyses suggest that the development of females and males already precedes the subadult stage. Within species, however, the same body parts can vary greatly in size between the sexes. Therefore, a complete morphological description of organ development can help identify the critical stages of female-male differential development. However, most studies have focused on the development of spider gonads only at mature or subadult stages and have only analysed the correlation of gonads in single-sex spiders (Choi and Moon, 2003; Michalik, 2009). This may be due to the external morphological differences between males and females being minimal at early stages, and sexing pre-subadult animals has proved difficult.

The SSD of spiders is usually studied based on single linear measurements of size, such as the maximum carapace width (Jakob et al., 1996). Since the size of carapace does not change with the physiological state and can stay fixed between moults, it is usually used to represent the body size of a spider (Prenter et al., 1995). On the contrary, the abdomen expands as a spider feeds, so it is not usually used as a reference for body size. The abdomen is most associated with egg production and storage, it can be used to estimate reproductive ability, which is closely linked to SSD. In addition, a study of the nephilid spiders revealed that genital size and somatic size are components of SSD (Lupše et al., 2016). In mammals, hormones secreted by the gonads trigger the development of dimorphism (Wilhelm et al., 2007). Other research about *Drosophila* has shown differences in the timing of gonad development between females and males and how this timing is regulated in a sex-specific manner is worth exploring (Whitworth et al., 2012). However, SSD is a multifaceted phenomenon, and there are limitations to using a single trait to generalize this complex and crucial biological problem. The combination of body size and gonad development in spider SSD has not been characterized in detail.

Allometric growth refers to the relationship between organs and body size (Mirth et al., 2016). Changes in allometric growth have resulted in a vast diversity of organism shapes. The difference in organ to body scale between sexes results in diverse morphologies, leading to SSD (Nijhout et al., 2014). A previous study demonstrated that female and male genital size in arthropods showed allometric traits (Eberhard, 2009). However, these studies have rarely discussed allometric development between the sexes in spiders. For spiders, body and gonadal size are evolutionarily decoupled (Ramos et al., 2005). From a metabolic point of view, however, both growth and development are costly. Thus, females and males may trade off body size and gonad size due to reproductive roles (female egg-laying capacity and male competition) and lifestyle (wandering). Mature individuals do not moult and their sclerotized bodies impede further growth. Therefore, reproductive organs and body size were determined by the growth stage of spiderlings. Comparing the growth trajectories of diverse morphologies can identify specific developmental periods, thus pointing to the developmental mechanism of spider morphologies between the sexes.

In the present study, we investigated the sex-specific growth strategies which were considered as the basis for studying SSD. We used a wolf spider, *Pardosa pseudoannulata,* to consider the body growth and gonad development between the sexes. *P. pseudoannulata* is a common polyphagous predator in rice fields in China (Preap et al., 2001). It is a popular laboratory animal (Li et al., 2016; Yu et al., 2020), and its biology is well understood (Iida and Fujisaki, 2005). In addition, it is less affected by food restrictions and environmental changes than other spider species and the female body size is larger than the male body size, so *P. pseudoannulata* is a particularly suitable model for SSD research. We tested for the size of the carapace, abdomen, and the area of gonads of spiders in different instars, from hatching to maturity, among the sexes to reveal the detailed ontogeny of SSD. We expect the organ size to scale with the size of the body parameters leading to allometric growth, which can elucidate the developmental processes that regulate the growth of organs and body parts. We tested the hypothesis that there are sex differences in allometric growth of the carapace, abdomen, and gonads between females and males.

## RESULTS

### Growth trajectories

**Fig. 1.**
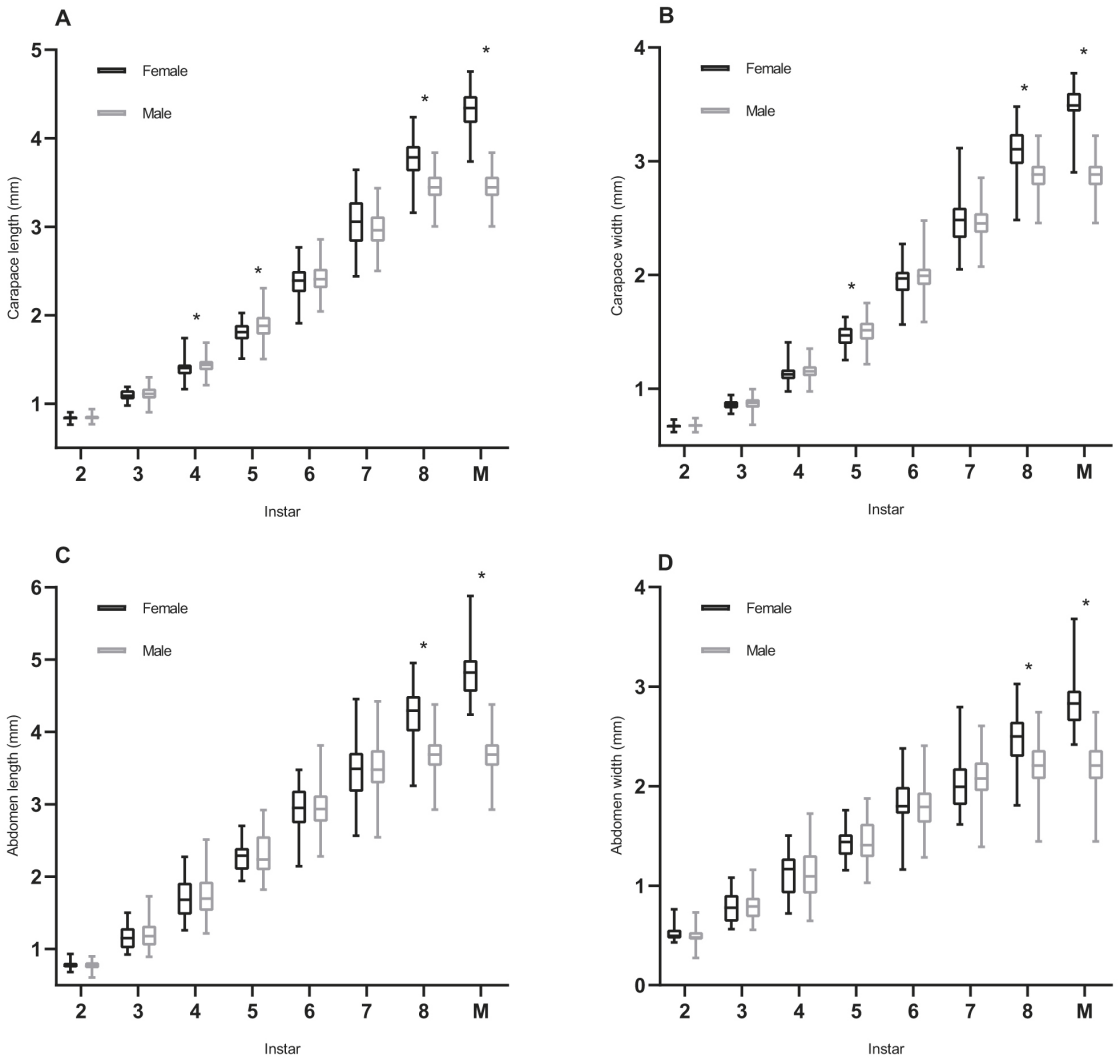
**Effect of sexes and instar on body size of *P. pseudoannulata* during development.** (A) Carapace length; (B) carapace width; (C) abdomen length; (D) abdomen width. M, mature. Box plots show median (horizontal line) values, upper and lower quartiles (box), and the minimum and maximum values (whiskers); asterisk (*) indicates a significant difference between females and males (*P*<0.05); females, *N*=28; males, *N*=84.

Instar and sex had a significant effect on spider carapace size [sex: *t*=3.297, *P*<0.001; instar: *t*=114.511, *P*<0.001 (length) and sex: *t*=2.518, *P*<0.05; instar: *t*=127.284, *P*<0.001 (width)] (Table 1). Meanwhile, there was significant interaction between instar and sex on carapace size. Mother identity and spider identity as random effects had no significant effect on carapace size (Table 1). Females and males differed significantly in the carapace length at the fourth instar (*U*=866.5, *P*<0.05), fifth instar (d.f.=110, *t*=−2.333, *P*<0.05), and eighth instar (d.f.=33.88, *t*=5.815, *P*<0.001) (Fig. 1A). Carapace width at the fifth instar (d.f.=110, *t*=−2.194, *P*<0.05) and eighth instar (*U*=489.5, *P*<0.001) (Fig. 1B) were significantly different between the sexes. Abdomen size [sex: *t*=1.347, *P*=0.178; instar: *t*=37.159, *P*<0.001 (length) and sex: *t*=0.619, *P*=0.536; instar: *t*=37.652, *P*<0.001 (width)] was significantly influenced only by the instar (Table 1). Similarly, mother identity and spider identity had no significant effect on abdomen size (Table 1). Unlike carapace size, the significant difference in the abdomen size between females and males occurs at the eighth instar (length: d.f.=34.644, *t*=6.839, *P*<0.001; width: d.f.=110, *t*=5.156, *P*<0.001) (Fig. 1C,D). The analysis of body size of mature females and males showed that there were significant differences in carapace size (length: d.f.=110, *t*=23.304, *P*<0.001; width: *U*=37, *P*<0.001) and abdomen size (length: *U*=2, *P*<0.001; width: *U*=48, *P*<0.001) between the sexes (Fig. 1A,B,C,D). However, there were no significant differences in body size between the sexes at other instars.

**Table 1.**
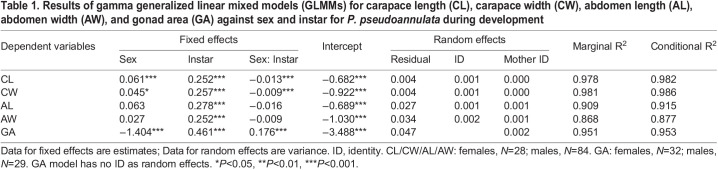
Results of gamma generalized linear mixed models (GLMMs) for carapace length (CL), carapace width (CW), abdomen length (AL), abdomen width (AW), and gonad area (GA) against sex and instar for *P. pseudoannulata* during development

The growth rate of carapace size differed significantly between the sexes from the sixth to the seventh instars (I_6–7_) (length: d.f.=34.491, *t*=2.299, *P*<0.05; width: d.f.=35.976, *t*=2.238, *P*<0.05) (Fig. 3A,B) and the seventh to the eighth instars (I_7–8_) (length: d.f.=110, *t*=5.119, *P*<0.001; width: *U*=402.5, *P*<0.001) (Fig. 3A,B). From the seventh to eighth instars, the growth rate of abdomen size in females was significantly higher than that in males (length: *U*=267.5, *P*<0.001; width: *U*=575, *P*<0.001) (Fig. 3C,D). The other comparisons did not yield significant results.

Developmental duration of females was not significantly different from that of males except for the sixth to seventh instars (*U*=798.5, *P*<0.05) and the seventh to the eighth instars (*U*=888.5, *P*<0.05) (Table 2). Before the eighth instar, the total developmental duration of males was longer than that of females (*U*=8575, *P*<0.05). Throughout the ontogenetic stage, females grow an extra moult compared to males.

**Table 2.**
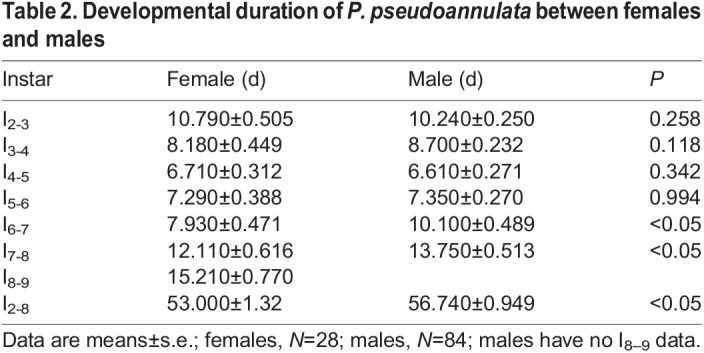
Developmental duration of *P. pseudoannulata* between females and males

Carapace length showed positive allometry in relationship to abdomen length in the ontogenetic stage, corresponding to slopes ranging from 0.109 to 0.667 and intercepts ranging from −0.149 to 1.069 (Fig. 4). Females and males differed significantly in their fifth instar (ANCOVA, *P*<0.05) (Fig. 4D), seventh instar (ANCOVA, *P*<0.05) (Fig. 4F), and eighth instar (ANCOVA, *P*<0.05) (Fig. 4G) allometric slopes.

**Fig. 2.**
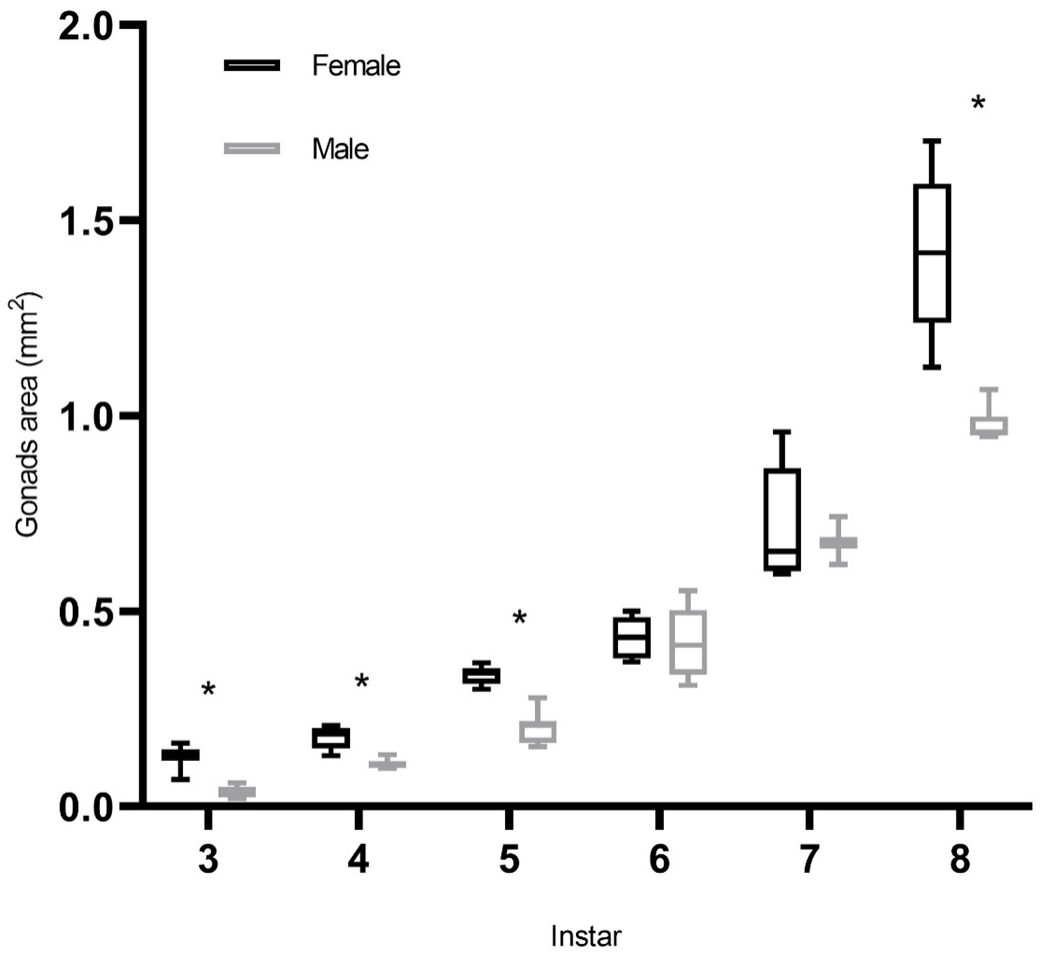
**Effect of sexes and instar on gonad area of *P. pseudoannulata* during development.** Box plots show median (horizontal line) values, upper and lower quartiles (box), and the minimum and maximum values (whiskers); asterisk (*) indicates a significant difference between females and males (*P*<0.05); females, *N*=32; males, *N*=29.

**Fig. 3.**
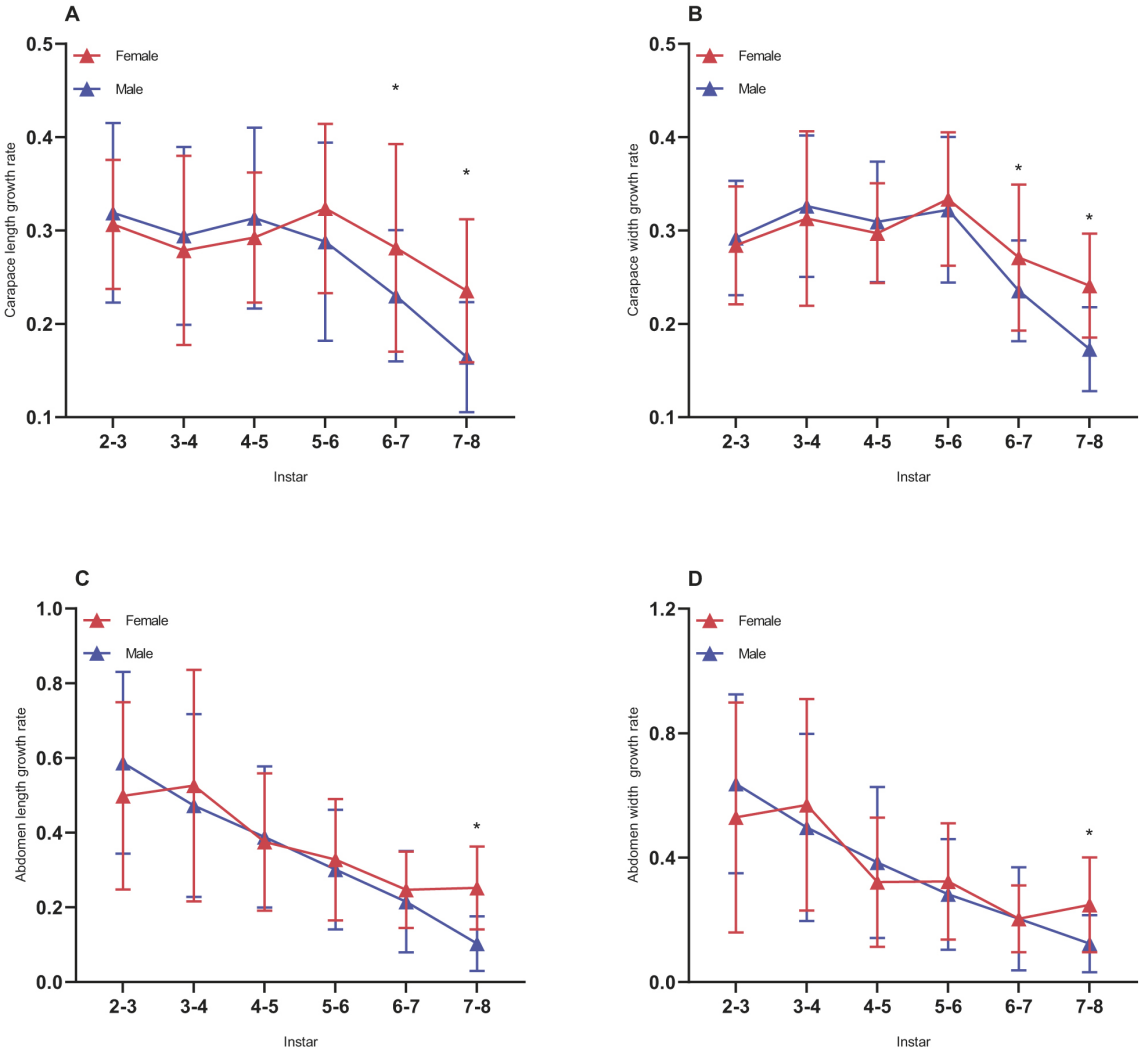
**Growth rate of body size of *P. pseudoannulata* during development between females and males.** Growth rate of (A) carapace length, (B) carapace width, (C) abdomen length, and (D) abdomen width of spiders from the six stages. Whiskers correspond to the range; asterisk (*) indicates a significant difference between females and males (*P*<0.05); females, *N*=28; males, *N*=84.

**Fig. 4.**
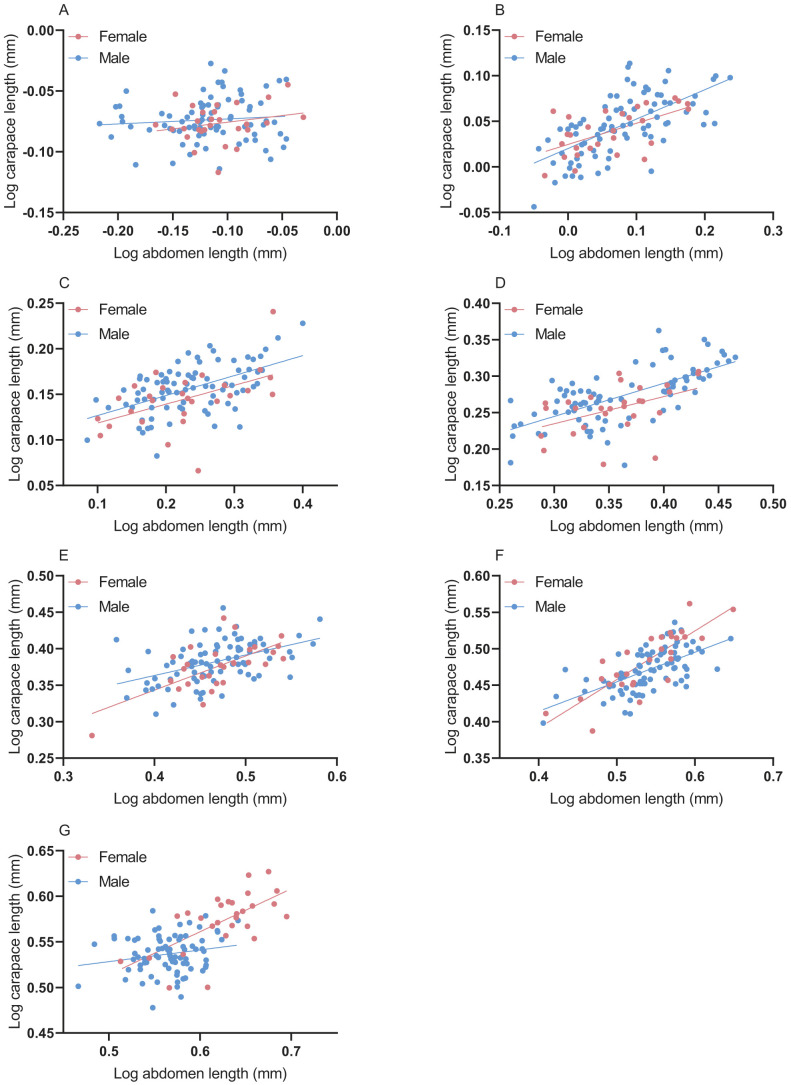
**Carapace-abdomen size allometry across ontogeny of *P. pseudoannulata* between females and males.** (A-G) Carapace-abdomen size allometry of *P. pseudoannulata* between females and males from second instar to eighth instar.

### Gonad development

Instar, sex, and their interaction affected the gonad area of spiders (sex: *t*=−9.016, *P*<0.001; instar: *t*=19.287, *P*<0.001) (Table 1). Mother identity as a random effect had no significant effect on gonad area. The gonad area of spiderlings at the third instar (d.f.=16, *t*=9.189, *P*<0.001), fourth instar (*U*=1, *P*<0.001), fifth instar (d.f.=14, *t*=7.886, *P*<0.001), and eighth instar (*U*<0.001, *P*<0.001) differed significantly between females and males (Fig. 2). Differences between the sexes at other stages were not statistically significant.

**Fig. 5.**
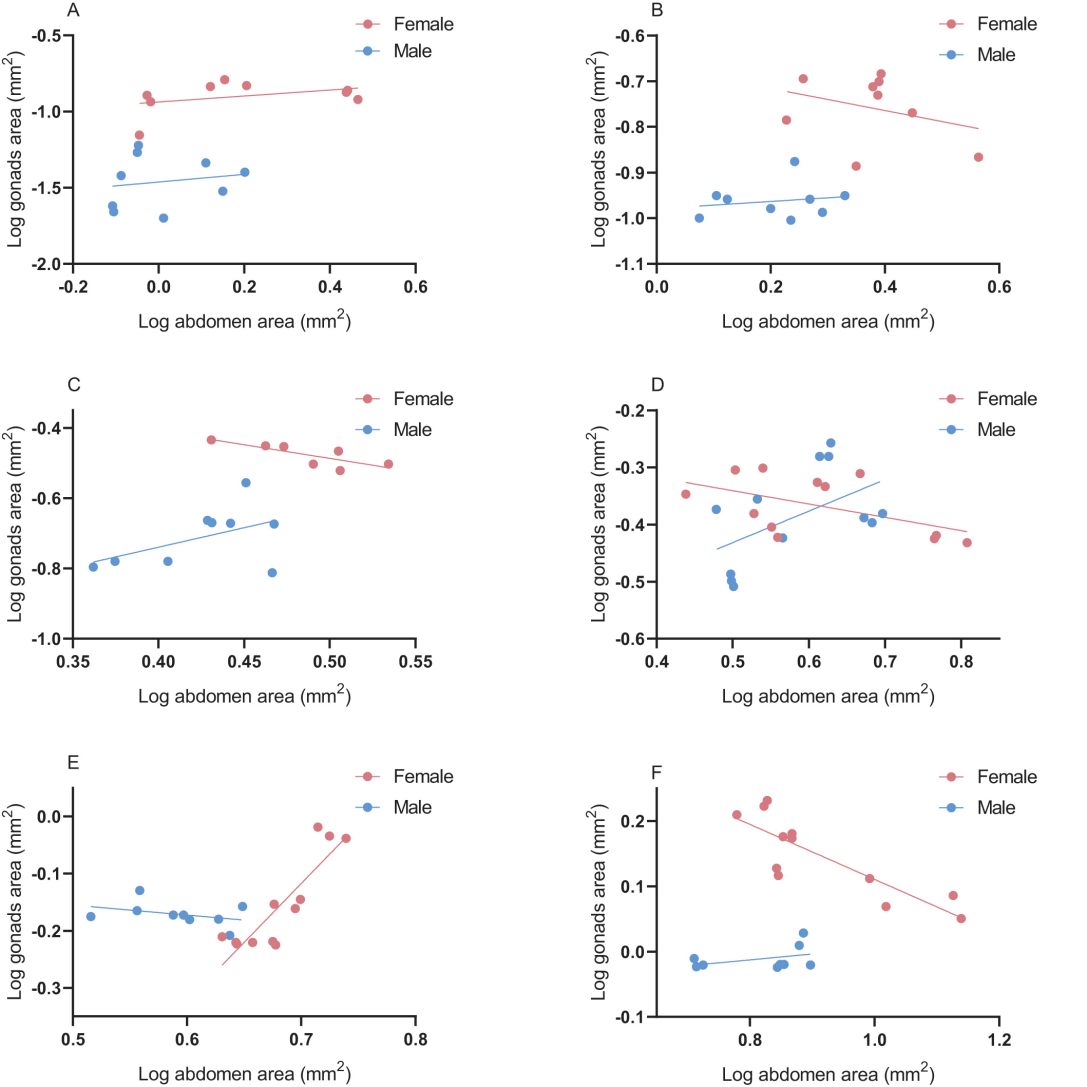
**Gonad-abdomen area allometry across ontogeny of *P. pseudoannulata* between females and males.** (A-F) Gonad-abdomen area allometry of *P. pseudoannulata* between females and males from third instar to eighth instar.

Positive and negative allometric developments of gonads and abdomen appeared in different instars. Both allometric parameters vary in the ontogenetic stage and corresponded to slopes ranging from −0.776 to 2.065 and intercepts ranging from −3.600 to 1.224 (Fig. 5). When spiders were at the third instar (ANCOVA, *P*<0.001) (Fig. 5A), fourth instar (ANCOVA, *P*<0.001) (Fig. 5B), fifth instar (ANCOVA, *P*<0.05) (Fig. 5C), and eighth instar (ANCOVA, *P*<0.001) (Fig. 5F), all slopes are significantly different between the sexes. The allometric intercepts of gonads and abdomen were different at the sixth instar (ANCOVA, *P*<0.05) (Fig. 5D) and seventh instar (ANCOVA, *P*<0.001) (Fig. 5E).

**Fig. 6.**
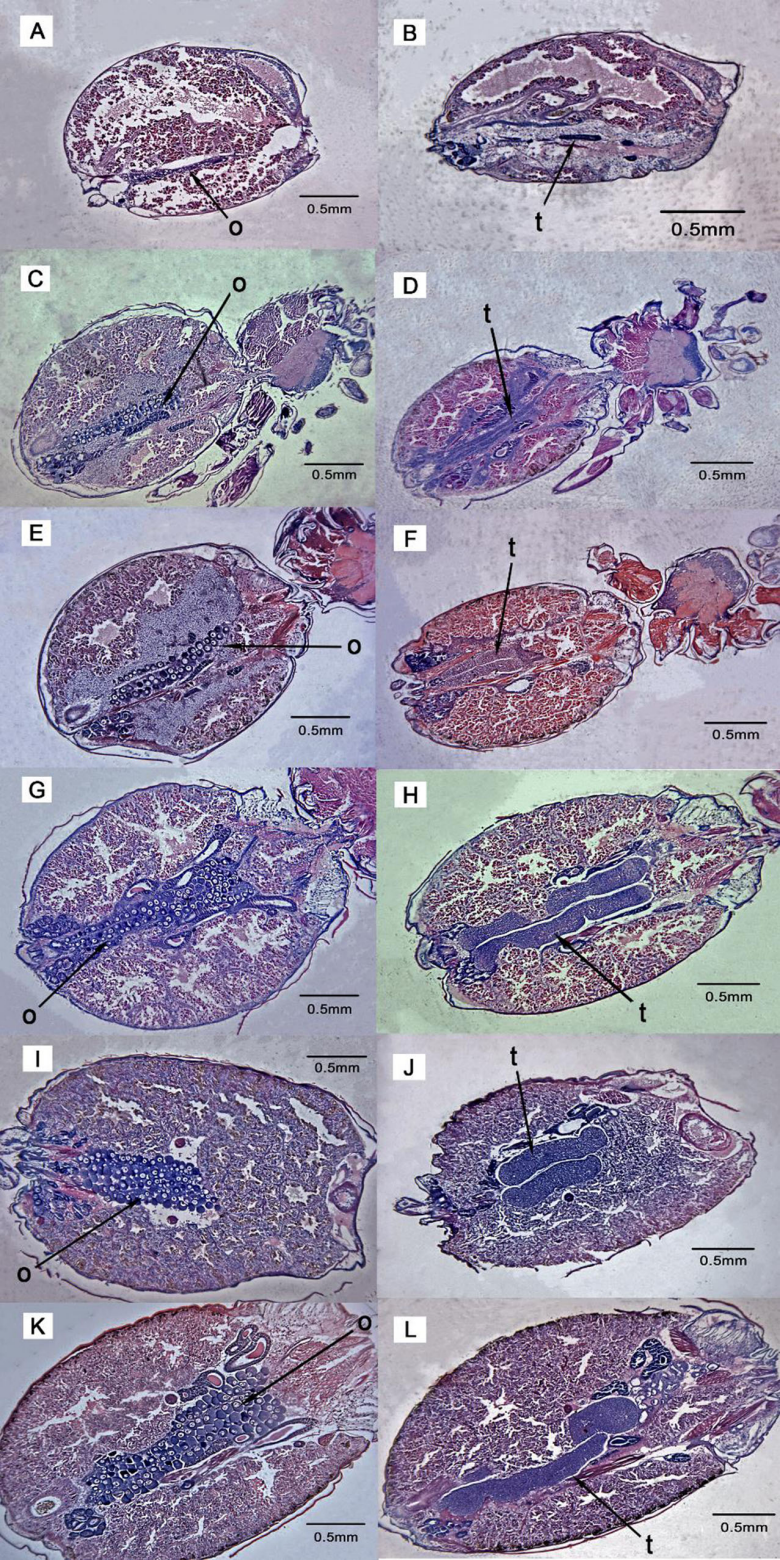
**Micrographs of abdomens of females and males of *P. pseudoannulata*.** Photo shows development of female ovary (o) (A,C,E,G,I,K) and male testes (t) (B,D,F,H,J,L) from third instar to eighth instar*.*

The ovary of the female spider has paired organs, longitudinally situated in the abdomen. Ovarian epithelium and numerous developing oocytes compose the ovary, which appears like two clusters of grapes. Using the scanning micrograph of the ovary from the third instar to the eighth instar, the proportion of ovary in the abdomen was gradually increased in size (Fig. 6A,C,E,G,I,K). The oocyte development was clearly not synchronous. At the early stages, oocytes were few in number and small in size, and the nucleus and the nucleolus were prominent. As the instar changes, the protein yolk was rapidly formed and the oocytes increase in size and number. During ovarian maturation, the nucleocytoplasmic ratio decreased. From the photograph, we observed that the density of oocytes rapidly increased from the sixth instar (Fig. 6G) to the seventh instar (Fig. 6I) and densely cluster in the abdomen. The male testes of the spider consist of paired tubular structures, extending deep into the abdominal cavity and lying parallel to the median axis (Fig. 6B,D,F,H,J,L). The lumen of the testes was filled with spermatozoa. The micrograph of the testes shows that the dimensions of the testis at the early instar are quite different from the final dimensions in adult males. From the fifth instar (Fig. 6F) to the sixth instar (Fig. 6H), the testes grow rapidly in dimensions.

## DISCUSSION

Understanding SSD requires knowledge of how SSD was formed during growth and development (Badyaev, 2002). However, few studies on sexual size dimorphism have focused on when size differences arise during ontogeny. Using integrative analysis, we show that sex differences of *P. pseudoannulata* as implied by the variations of body growth and gonad development between females and males. The gonad differences between the sexes are apparent early in the life cycle. Our study supports the hypothesis that allometric growth occurs in the carapace, abdomen, and gonads, and there are sex differences in allometric growth between females and males.

Sex differences are shown between the sexes of *P. pseudoannulata*, but the differences are moderate. Terrestrial arthropods show a slightly to moderately female-biased SSD. Although some female-biased extreme SSD have been reported in spiders (Kuntner and Coddington, 2020), moderate SSD are a general pattern in wolf spiders (Vollrath and Parker, 1992). The moderate SSD is due to the sex-specific developmental processes of the *P. pseudoannulata* (Stillwell and Davidowitz, 2010). In our study, one part of the body-the carapace-was affected by sex, while the abdomen was not. Body growth of females and males is similar throughout ontogeny, except for the difference in body size between the sexes at some instars. Sexual dimorphic organs, like the ovary and testes, used for gamete formation are often the most obvious manifestations of sex differences (Bell, 1982). In this study, the area differences of female and male gonads were varied with different instars. Thus, moderate SSD was driven by instars and body part variations in body size and gonad area differences between the sexes in *P. pseudoannulata.*

Sex differences of *P. pseudoannulata* come up early in life and reappear in the subadult stage. There are only three ways that females and males can become different in body size during development: the sexes must differ in their size at hatching, growth rate, and/or developmental duration (Stillwell et al., 2010). We did not observe any difference in body size between females and males at hatching. The relationship between body size and sex varies depending on the instar of spiders. Differences of growth rate between the sexes are not invariant or linear throughout development. In the ontogenetic stage, the developmental duration difference between females and males is in a state of fluctuation. Although there is no difference in the developmental duration between the sexes at the fifth instar, females grow faster than males from the fifth instar. Therefore, there is a significant difference in carapace size between females and males at this stage. In addition, our data analyses showed that the difference in body size between the sexes appeared in later instars. From the perspective of developmental duration, in the last instar before sexual maturation, males increase their body size by extending the number of developmental days in the instar. Differently from males, females prolong their growth period by moulting once more than males and thus increasing their size (Esperk et al., 2007; Kuntner et al., 2012). In terms of growth rate, females grow faster than males in later instars. Previous studies have shown that there is plasticity in growth strategies between the sexes (Kleinteich and Schneider, 2010). Growth plasticity can be achieved by females delaying maturation, and males can optimize body size when necessary (Head, 1995). This means SSD may be directly caused by differences in energy absorption and dissipation between females and males. In other words, the larger sex may forage more efficiently than the smaller one at all stages (Rohner et al., 2018). The combination of these factors partly explains female-biased SSD of *P. pseudoannulata.* These results indicate that females and males have different growth states in the same instar.

*P. pseudoannulata* showed morphological changes in the gonads to varying degrees during different instars. Different from the development of body size, the area of the ovary and testes was significantly different at hatching. One possibility is that spiders, as heterogamous species, have larger female gametes and small male gametes (Cordellier et al., 2020). The testes area increased rapidly from the fifth instar. From the sixth instar on, however, the ovaries develop rapidly until they mature beyond the testes. Interestingly, this finding suggests that gonad development in males started one instar earlier than in females, matching the finding that females have one more instar than males to reach maturation. Therefore, the difference in gonad size between females and males was realized by the difference in size at hatching, the speed of growth rate, and the length of developmental duration. SSD of the gonads is essential for the production of female and male gametes required for sexual reproduction. The cost of sperm production is much lower than that of eggs. Male gametes are usually optimized for high mobility, while female gametes are usually optimized for nurturing the zygote after fertilization (Bulmer and Parker, 2002). There is growing evidence that hormones secreted by the gonads can modify growth and may contribute to the development of SSD (Cox et al., 2009; Starostova et al., 2013). We speculate that gonadal secretions regulate the close temporal correspondence between sexual divergence in growth rate and gonadal differentiation to establish SSD (Hayes and Licht, 1992). The ways in which gonad secretions regulate differences between the sexes require further study. These results suggest the rate of gonad development is not constant or linear between the sexes throughout development.

Sex differences showed in allometric growth of body parameters between females and males of *P. pseudoannulata*. We observe that body proportions are generally not constant throughout the ontogenetic stage. The change of gonad area was not consistent with the variety in abdomen size during the whole ontogeny. Changes of these measured values reflect changes in resource investment in various parts of the body (Iida and Fujisaki, 2007). The results of the present study clearly show that instar changes in resource allocation occur among the carapace, abdomen, and gonads. We found that investment in carapace development increases from the hatching period then the investment in abdomen growth increases at the fourth instar compared with the third instar. Previous research indicated that the spider carapace relates to locomotion, food intake, and integrative nervous functions. The abdomen of spiders relates to vegetative tasks, including reproduction (Foelix, 1996). Thus, when hunting ability is important for the more vulnerable hatchling, increasing resource allocation to develop the carapace increases its size. Similarly, increased abdomen resource allocation during the rapid-development stage enhances hunger tolerance and provides reserves for reproduction (Iida and Fujisaki, 2007). Spiders that invest more in carapace development from the fifth instar also invest more in gonad development. Since gonad development is strictly controlled by neurohormones (Bednarek et al., 2019) and the carapace is associated with integrative nervous functions, the carapace and gonads have similar developmental trends. As the part of the body where gonads are located, egg and sperm loads contribute significantly to the abdomen in spiders (Prenter et al., 1994; Wickman and Karlsson, 1989). In other words, abdominal size restrains the space available for storing egg-making or ejaculate materials and egg-producing or ejaculating organs (Wickman and Karlsson, 1989). After the gonad development investment increased, the proportion of investment in abdomen development also increased. Thus, the size variation in organ and body parameters arises from developmental plasticity.

It is also interesting to note that morphology scales differently between the sexes at the fifth instar (slopes different for females and males), and scales of gonads to abdomen differ between the sexes at this instar. This discrepancy is due to the fact that male gonads develop earlier than females’, and the earlier development of external morphologies than internal organs. The carapace–abdomen allometries in females differed from those of males later in ontogeny. This can be explained by the different allocation of resources between the sexes in the subadult stage. Males need to invest more in the carapace than other body parts because a larger carapace can accommodate the increased muscle tissue for maximum protection from cannibalism (Iida, 2003) and acts as an advantage in agonistic mating (Prenter, 1992). As a predictor of fertility before mating (Head, 1995) and the part of fertilized egg after fertilization (Bulmer and Parker, 2002), the female abdomen has a higher priority in terms of somatic investment compared with other body parts. In both females and males in the subadult stage, the proportion of gonads investment relative to the abdomen is reduced, likely when the growth of the organ stops or slows down while the increase in body size is sustained or accelerates. These may be related to the increase of endocrine hormones in arthropods in one instar prior to adulthood (Chelini et al., 2019). The trade-off hypothesis holds that gender segregation has major advantages because costs can be allocated efficiently between the two genders. The gonads of males develop earlier than females, allowing male spiders to redistribute energy to other parts of the body for maturation and engage in mating strategies earlier (Dodson et al., 2015). Early maturity also reduces male juvenile mortality, which in turn reduces the mortality rate of mature males, skewing the mature spider sex ratio (Vollrath and Parker, 1992). Compared with males, female optimum depends on how size increases fecundity, so they are more dependent on resources collected by spiderling and stored in the abdomen. Females adapt to the selection pressure with a growth strategy of increasing body size at longer juvenile life (Vollrath and Parker, 1992) and having larger gonads early in life. As individuals grow, females and males allocate resources differentially to body parameters or organs to ensure that the size of morphological traits matches the final body size. This will result in different allometric growth patterns (Zelditch et al., 2012), which would lead to SSD (Fairbairn, 2007).

In conclusion, there is a moderate SSD between females and males of *P. pseudoannulata.* This long-term study indicates developmental differences between females and males happens in early stages and throughout the entire ontogeny*.* The differences in growth rate and developmental duration between the sexes change nonlinearly at the ontogenetic stage. In addition, allometric growth was found in the carapace, abdomen, and gonads of female and male spiders. Allometric growth describes the scales of organs to body in ontogenesis, capturing the relationship among the body parts. Moreover, we provide a complete morphological description of gonad development. It will be helpful to better understand moderate SSD and the underlying developmental mechanism of *P. pseudoannulata*. Together with an understanding of the developmental plasticity processes of organ and body size, this provides a powerful entryway into understanding how allometry of SSD evolved for spiders.

## MATERIAL AND METHODS

### Spider rearing

We collected 32 female *P. pseudoannulata* carrying egg sacs from rice fields at Huazhong Agricultural University in Wuhan, Hubei Province, China (30°52′N, 114°31′E) in September 2019. The female spiders were housed individually in glass tubes (diameter 2 cm, length 10 cm) under a 14 h:10 h light: dark photoperiod at 25°C and with 50–70% relative humidity in the laboratory. A piece of water-dampened sponge was placed at the bottom of each glass tube to provide water for the spider, and the tube was plugged with cotton wool. We alternately fed the spiders with *Drosophila melanogaster* and *Tendipes sp*. every 3 days until spiderlings emerged from the egg sacs. The spiderlings emerged and we selected them from egg sacs and kept each juvenile in individual glass tubes (diameter 2 cm, length 6 cm). We provided them the same environmental conditions and food regime as their mothers. The developmental status (moulting) of the spiderlings was checked three times per week. The spiderlings were used for the following experiments.

### Growth trajectories

We selected all spiderlings (*N*=237) and followed their growth trajectories. The spiderlings (*N*_1_=70, *N*_2_=105, *N*_3_=62) were derived from three egg sacs which were carried by three randomly selected females. On the day when spiderlings emerged as the second instar, we measured the lengths and widths of their carapace and abdomen. Emerging spiderlings were considered the second instars as they underwent the first moult within the egg sacs (Peck and Whitcomb, 1970). We also measured the same body size parameters (carapace length and width; abdomen length and width) for each spiderling after each moult in the subsequent instars until they reached adulthood. Photographs were taken with a Leica DFC495 digital camera mounted on a Leica M205C (Leica Microsystems GmbH, Wetzlar, Germany) compound microscope then measurements were performed under its supporting software LAS Application Suite V4.3 to the nearest 0.01 mm. There were 112 spiderlings in this study that developed to adulthood, where females matured in the ninth instar (*N*=28) and males matured at the eighth instar (*N*=84). We can only identify the sex of spiderlings at the seventh instar (subadult stage of the male), and most of the dead spiderlings’ sex at early instars was unknown. We collected data on the body size of all the spiders that survived to maturity. Developmental duration (from the second instar to mature) for each instar of each spider was recorded.

### Gonad development

We selected all instars (second–ninth) of spiders (*N*=240) as specimens, which were born in the laboratory. The specimens were the offspring of 13 females, and 30 spiders were randomly selected from each instar in laboratory reading. The abdomens of these specimens were sliced and observed by a light microscope. Spiders at different instars were anesthetized with carbon dioxide and fixed in Carnoy fixative solution for 4 h. The specimens were dehydrated in ethanol for 2 h then fixed in benzoic acid methyl ester overnight at room temperature. After being soaked in the mixed solution (benzoic acid methyl ester and paraffin) at 63°C for 1 h, they were infiltrated with paraffin (total of eight times, each 20 min) and finally embedded in paraffin wax. The embedded blocks were sliced into 8-µm-thick sections by rocking microtome and mounted on slides. To ensure adhesion to the slide, sections on slides were heated in an oven at 58°C overnight. Sections were dewaxed three times in xylene, each for 15 min, followed by rehydration through a graded series of ethanol. Sections were then stained by haematoxylin-eosin (HE) solution and fixed onto permanent microscopy slides. We collected images from 2102 slices of 61 *P. pseudoannulata* specimens (*N*=32 for females and *N*=29 for males). All sections from three females and three males for each instar stage (except for the second instar as we could not get the sections of gonad in this instar) were photographed using a Canon G10 digital camera (14.7 megapixels) mounted on an Olympus BX51 compound microscope. We selected three sections containing the largest area of gonad from a single specimen to investigate the details and measured the area of gonads and the abdomen using ImageJ. There were 118 sections from 21 females and 19 males at different instars to evaluate differences between the sexes with regard to the development of *P. pseudoannulata*. The photograph of the section containing the ovary or testis was used to distinguish between female and male.

### Statistical analysis

We used two gamma generalized linear mixed models (GLMMs) with a log link to evaluate differences between the sexes. In the growth trajectories model, we used the instar and sex as predictor variables, body size of each spiderling at each instar as the response variable, and the mother identity with spider identity as a random effect. In the gonad development model, we used the same independent variables as described above and used gonad area as the response variable. Gamma distribution best fits the data of body size and gonad area for our models (Payne, 2014). Differences in development of body size or gonad area for total stages (except for the ninth instar because most males matured in the eight instars) between females and males in the same instar were performed using the independent-samples *t*-test or the Mann–Whitney *U*-test. We used the same test to analyse the body size differences between mature female and male spiders. We did not test gonads of the second instar spiderlings because they were too small to obtain a complete section. We used the Shapiro–Wilks test to check the normality of the body size or gonad area before analyses.

To determine the growth rate at different stages between the sexes, we calculated the growth rate {growth rate=[body size_(I+1)_-body size_(I)_]/body size_(I)_, I=instar} of spider body size in the growth trajectories treatment group. The Shapiro–Wilks test was used to evaluate the data normality. We used independent-samples *t*-test or the Mann–Whitney *U*-test to test the differences in growth rate and developmental duration between the sexes. Since most of the males were not developed to the ninth instar, we did not analyse growth rates and developmental duration from the eighth instar to the ninth instar.

To explore carapace and abdomen allometry during the ontogeny, all measurements of carapace length and abdomen length were logarithm transformed for linearity and assessed using ordinary least squares (OLS) regression. The allometric growth relationship between gonads and abdomen were also analysed using the same method. We use analysis of covariance (ANCOVA) to examine how the relationship differs from isometry for each sex. The Shapiro–Wilks test and Fligner–Killeen test were used to check the normality and homoscedasticity of the data before analysis. Similarly, we did not analyse the differences in allometry between females and males at the ninth instar. Allometric analysis of the gonads and abdomen also excludes the second instar spiderlings.

We ran GLMMs analyses in R version 4.0.3 (R Core Team, 2020), using the *glmer* function in the *lme4* package (Bates et al., 2015). We ran OLS regression analyses using R's package *lme4*. We used R's package *multcomp* for ANCOVA regression analysis. The statistics of independent-samples *t*-test and the Mann–Whitney *U*-test analyses were performed with SPSS (version 16.0; SPSS Inc., Chicago, IL, USA).
